# Intra-articular injection choice for osteoarthritis: making sense of cell source—an updated systematic review and dual network meta-analysis

**DOI:** 10.1186/s13075-022-02953-0

**Published:** 2022-11-28

**Authors:** Yijian Zhang, Huilin Yang, Fan He, Xuesong Zhu

**Affiliations:** 1 Department of Orthopaedics, The First Affiliated Hospital of Soochow University, Soochow University, No. 899 Pinghai Road, Suzhou, 215006 China; 2 Orthopaedic Institute, Medical College, Soochow University, No. 708 Renmin Road, Suzhou, 215007 China

**Keywords:** Mesenchymal stem cells, Osteoarthritis, Intra-articular injection, Pain relief, Function improvement, Adverse effects

## Abstract

**Background:**

Intra-articular injection is indicated for mild or moderate osteoarthritis (OA). However, the superiority of cell-based injection and the role of diverse cell sources are still unclear. This study aimed to compare the therapeutic effect of intra-articular injection with mesenchymal stem cells (MSCs) and cell-free methods for OA treatment.

**Methods:**

A literature search of published scientific data was carried out from PubMed, MEDLINE, Embase, Cochrane Library, Web of Science, and China National Knowledge Internet (CNKI). Randomized controlled trials (RCTs) compared the efficacy and safety of MSC and cell-free intra-articular injection treatments for OA with at least 6-month follow-up.

**Results:**

Dual network meta-analysis validated the therapeutic advantages of MSC treatments (VAS, Bayesian: 90% versus 10% and SUCRA: 94.9% versus 5.1%; WOMAC total, Bayesian: 83% versus 17% and SUCRA: 90.1% versus 9.9%) but also suggested a potential negative safety induced by cell injection (adverse events, Bayesian: 100% versus 0% and SUCRA: 98.2% versus 1.8%). For the MSC source aspect, adipose mesenchymal stem cells (ADMSCs) and umbilical cord mesenchymal stem cells (UBMSCs) showed a better curative effect on pain relief and function improvement compared with bone marrow mesenchymal stem cells (BMMSCs).

**Conclusion:**

Intra-articular injection of MSCs is associated with more effective pain alleviation and function improvement than cell-free OA treatment. However, the potential complications induced by MSCs should be emphasized. A comparative analysis of the MSC sources showed that ADMSCs and UBMSCs exerted a better anti-arthritic efficacy than BMMSCs.

**Graphical Abstract:**

Schematic illustration of MSC-based intra-articular injection for treating OA. Three major MSCs (UBMSCs, ADMSCs, and BMMSCs) are extracted and expanded in vitro. Subsequently, the amplified MSCs are concentrated and injected into the knee joint to treat OA.
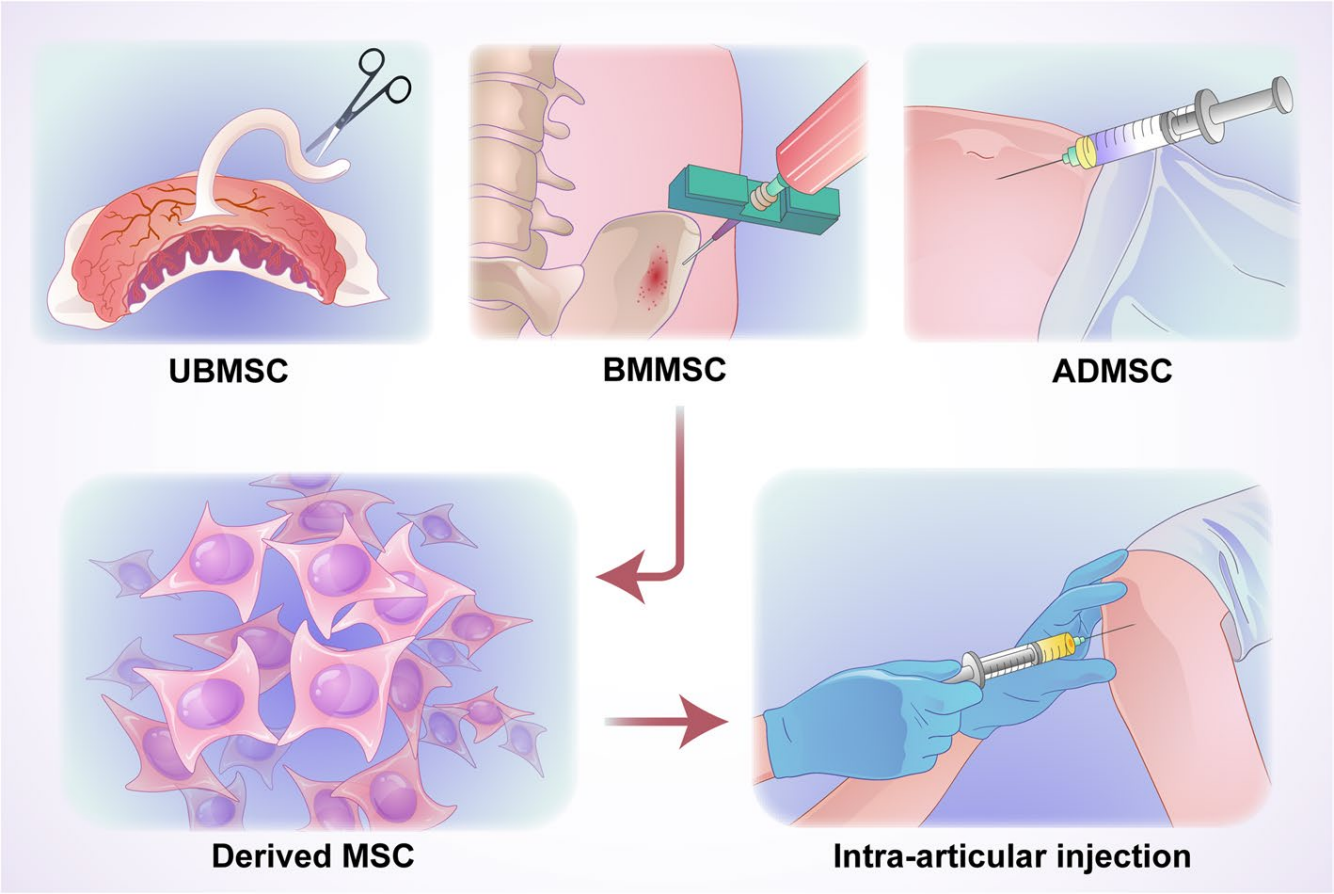

**Supplementary Information:**

The online version contains supplementary material available at 10.1186/s13075-022-02953-0.

## Key messages

1. Intra-articular injection of MSCs shows better improvements in pain and function than cell-free OA.
2. MSC-based therapy is associated with neglected treatment-related adverse event risk.
3. For the selection aspect, ADMSCs or UBMSCs may exert a better anti-arthritic efficacy than BMMSCs.

## Introduction

Osteoarthritis (OA) is a degenerative disorder of common joints such as the knee, hip, shoulder, and ankle. With a rising aging population, OA has led to immeasurable health and financial burden because it mainly affects elderly people [1]. Several therapeutic methods, e.g., oral nonsteroidal anti-inflammatory drugs (NSAIDs), physical therapy, and intra-articular injection treatment, have been developed to alleviate pain in patients suffering from OA [2]. The articular cavity is insensitive to the systemic administration of drugs due to a limited supply of oxygen and blood. Therefore, a direct intra-articular injection is an effective strategy for drug delivery [3]. Clinical studies show that intra-articular delivery of hyaluronic acid (HA), a common super-lubricated molecule, can alleviate OA-induced pain and stiffness at a short- or mid-term follow-up [4]. Corticosteroid (CS), platelet-rich plasma (PRP), and clodronate are been proven to be candidate drugs for treating OA by reducing inflammation and relieving pain [5, 6]. However, the lack of bioactivity of the above reagents limits long-term efficacy.

Recently, in-depth research on regenerative medicine represented by mesenchymal stem cells (MSCs) has provided a novel therapeutic strategy for OA treatment [7]. MSC-based intra-articular injections have excellent histocompatibility and robust bioactivity to promote repairs of injured cartilage and cartilage regeneration through injected stem cells and secreted extracellular matrix (ECM) components [8]. Bone marrow mesenchymal stem cells (BMMSCs) achieve satisfactory clinical outcomes due to their convenient availability that enhances its wide application for OA injection therapy [9]. Furthermore, previous studies have reported that other MSC sources including adipose mesenchymal stem cells (ADMSCs) and umbilical cord mesenchymal stem cells (UBMSCs) also showed a better curative effect on OA when compared with cell-free strategy. Therefore, there is a potential advantage of MSCs in the treatment of OA [10, 11]. Nevertheless, few research is reported on the comparison of different types of MSCs and cell-free treatments.

This article reviewed the latest published research reports related to these therapies of OA. We focused on the comparison of efficacy and safety of cell-based and cell-free intra-articular injection therapies. A dual network meta-analysis based on Bayesian and SUCRA models was also performed to provide a more reliable reference for clinical injected therapy of OA. The latest research progress related to these therapies, the mechanism of function, and the adverse effects of them were summarized and concluded. According to the results, the application prospect of intra-articular injection was analyzed in the treatment of osteoarthritis.

## Materials and methods

### Study design

This study followed the preferred reporting items (PRI) for the systematic reviews and incorporated Network Meta-Analysis (PRISMA-NMA) for meta-analyses Additional File 2, [12].

### Eligibility criteria

**Fig. 1:**
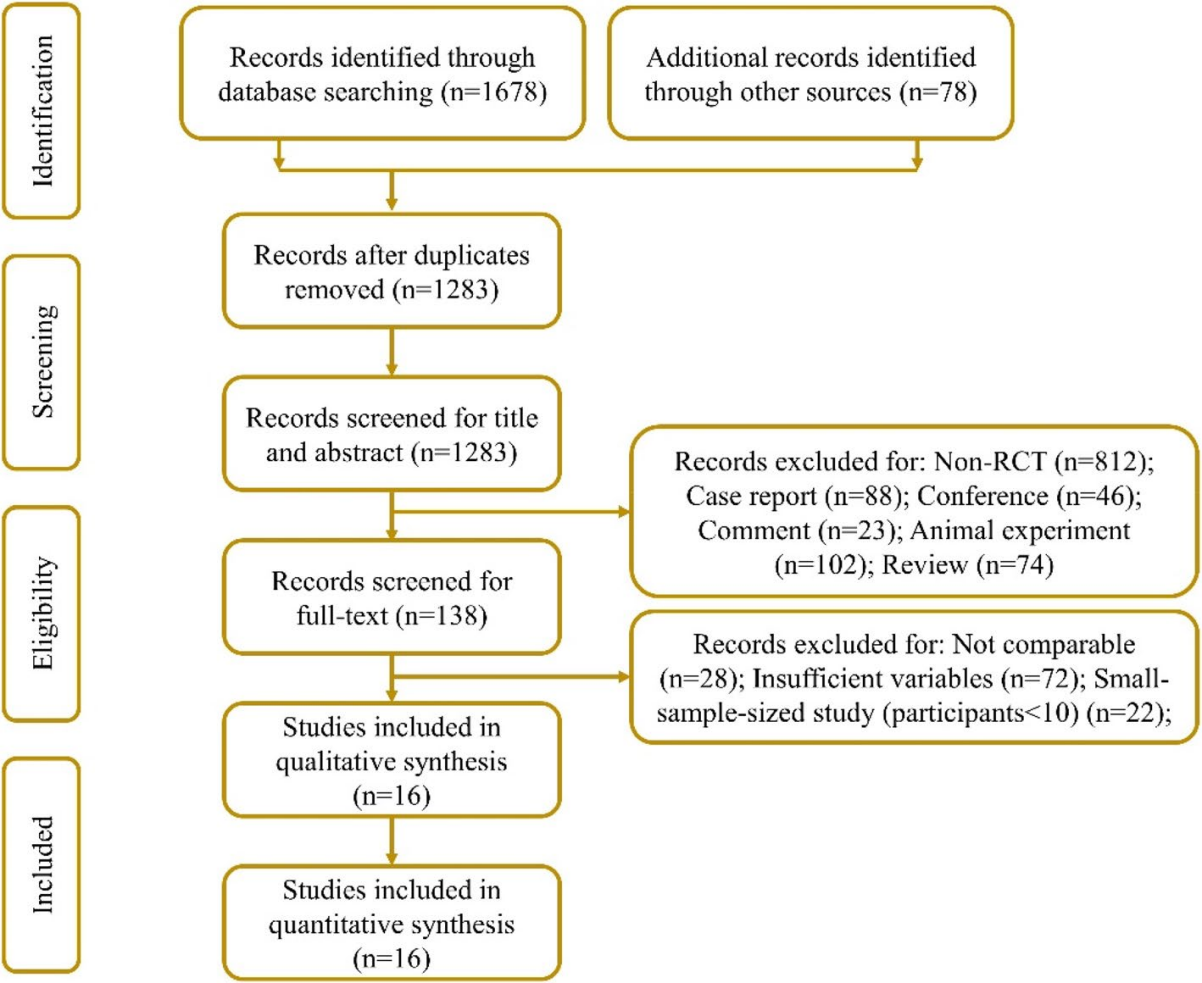
Selection flowchart of undertaken literature search. Databases include PubMed, Embase, Cochrane Library, MEDLINE, Web of Science, and China National Knowledge Internet (CNKI)

The inclusion criteria for enrollment of studies into network meta-analysis were participants 18 and above years of age; participants with unilateral or bilateral symptomatic knee OA (as described by the American College of Rheumatology criteria); visual analog scale (VAS); and Kellgren-Lawrence grade; participants who received an intervention of a single intra-articular injection with MSCs (BMMSCs, ADMSCs, or UBMSCs) and received a single intra-articular administration of PRP, HA, saline, or conservative treatment (CT, oral medication, etc.); and efficacy parameters including VAS scores, Western Ontario and McMaster Universities Osteoarthritis (WOMAC) index of the total, and safety indicators such as the incidence of adverse events and the study design of randomized controlled trials (RCTs) with a follow-up of at least 6 months. The exclusion criteria included non-RCT, case reports, conferences, comments, animal experiments, reviews, non-comparable studies, and studies with insufficient variables or a small sample size (included less than ten participants) (Fig. 1).

### Searching strategy

Public databases including China National Knowledge Internet (CNKI), Cochrane Library, Embase, MEDLINE, PubMed, and Web of Science were utilized to conduct a literature search based on the following algorithm: (“osteoarthritis” or “knee osteoarthritis” or “knee pain”) or (“mesenchymal stem cells” or “mesenchymal stromal cells” or “mesenchymal progenitor cell” or “stem cell” or “stromal cell” or “progenitor cell”) and randomized controlled trials. The reference lists of the obtained relevant review or studies were searched to prevent undiscovered omissions. The retrieval process was conducted from the date of inception to January 2021, and the language used was strictly English and Chinese.

### Data extraction

The extracted data included the authors, country, publication date, sample size, mean age, sex distribution, intervention details, outcome parameters, and follow-up time. The data were independently extracted by two authors (YZ and FH) and checked by a third reviewer (XZ). For efficacy index, VAS scores and WOMAC index of total, function, pain, or stiffness were collected pre- and post-intervention between the two arms. The incidence of adverse events (mild symptoms: mild arthralgia and joint effusion; moderate symptoms: moderate knee pain and swelling; severe symptoms: intense knee pain and acute synovitis) for the two groups was recorded after the intervention.

### Quality control

The Cochrane Collaboration Tool was used by the two independent reviewers (YZ and FH) to assess the methodological quality of each study enrolled for the analysis. According to this tool, “low,” “unclear,” or “high” risk were assigned to selection bias, detection bias, attribution bias, performance bias, reporting bias, and other biases.

### Statistical analysis

The Review Manager (v 5.3), STATA (v 14.0), WinBUGS (v 1.4), and R (v 3.6) software were utilized to perform data analyses following a prescribed order in this study. First, a conventional pairwise meta-analysis was conducted using random effects model with the STATA software (v 14.0). This estimated the relative risk (RR) for the dichotomous variables (with a 95% credible interval). The data from the continuous variables have a 95% credible interval. Second, network meta-analysis was performed based on a Bayesian random effects model using the GeMTC R package. The Markov chain Monte Carlo (MCMC) method was applied to obtain the pooled ranking probabilities. This was performed through 100,000 iterations for each three MCMC chains with a burn-in period of the initial 50,000 iterations. Third, to calibrate the reliability of the ranking probabilities, a SUCRA model [13] was generated from STATA. Furthermore, the pooled results from GeMTC and STATA were matched to provide a more credible conclusion. Ultimately, a series of tests were performed to confirm the stability of the pooled results. A loop test model was built based on STATA to determine the heterogeneity of each parameter. From this model, a moderate or high *I*^2^ value reflected a possible heterogeneity of the parameters. The global or local consistency was evaluated using an inconsistency model or node-splitting method, respectively, to determine the closed-loop parameters [14]. A gap between the consistency and inconsistency or between random effect variance and inconsistency variance was used to estimate the potential inconsistency of the open-loop variables [15].

## Results

### Study characteristics

A total of 1756 articles were identified and extracted from the different databases and literature sources. Out of the 1756 extracted articles, 1283 articles were included after the elimination of the duplicates. The 1283 studies remained for the title and abstract screening. Subsequently, the full-text review of 138 studies was conducted. Out of the 138 studies, 122 of studies were excluded according to the exclusion criteria. Finally, 16 RCTs with 612 patients were enrolled in this network meta-analysis (Fig. 1) [16–31]. The mean age of patients was 55.7 with a range from 51 to 60 years. The percentage of women included in the study was 40.6%. Among these eligible studies, 8 reported on the BMMSC treatment, 4 reported on the ADMSC treatment, and 4 reported on the UBMSC treatment. The overall mean follow-up time was 10.9 months with a range from 6 to 24 months. The specific information of each study was as presented in Table 1.

**Table 1:** Characteristics of the included studies

Study	Country	Trial number	Sample size	OA severity	Treatment process	Cell source	Mean age	Sex (M: F)	Intervention	Outcomes	Follow-up
Emadedin, 2018 [17]	Iran	NCT01504464	BMMSC = 19; saline = 24	KL of II, III, or IV; WOMAC > 25	IA of 5 mL	Autologous	53.4	27: 16	40 × 10^6^ BMMSCs	VAS, WOMAC^1~4^, AE	6 months
Freitag, 2019 [18]	Australia	ACTRN12614000814673	ADMSC = 10, CT = 10	KL of II or III; NRS > 5	IA of 3 mL	Autologous	53.1	12: 8	100 × 10^6^ ADMSCs	VAS, WOMAC^1^, AE	12 months
Gupta, 2016 [20]	India	NCT01453738	BMMSC = 10; HA = 10	KL of II or III	IA of 2 mL	Allogeneic	56.5	3: 17	25 × 10^6^ BMMSCs	VAS, WOMAC^1~4^, AE	12 months
Ha, 2018 [16]	China	–	UBMSC = 43; PRP = 44	KL of I, II, or III	IA of 5 mL	Allogeneic	56.3	26: 61	5 × 10^6^ UBMSCs	VAS, AE	12 months
Khalifeh, 2019 [21]	Iran	IRCT2015101823298N	UBMSC = 10; saline = 10	KL of II, III, or IV	IA of 10 mL	Allogeneic	56.7	2: 18	50 × 10^6^ UBMSCs	AE	6 months
Kuah, 2018 [22]	Australia	ACTRN12615000439549	ADMSC = 8; saline = 4	KL of I, II, or III; VAS > 35/100	IA of 2 mL	Allogeneic	52.2	5: 7	4 × 10^6^ ADMSCs	VAS, WOMAC^2^, AE	12 months
Lamo-Espinosa, 2016 [24]	Spain	NCT02123368	BMMSC = 10; HA = 10	KL of II, III, or IV; VAS > 2.5/10	IA of 4 mL	Autologous	63.1	11: 9	10 × 10^6^ BMMSCs	VAS, WOMAC^1~4^, AE	12 months
Lamo-Espinosa, 2020 [23]	Spain	NCT02365142	BMMSC = 24; PRP = 26	KL of II, III, or IV; VAS > 2.5/10	IA of 8 mL	Autologous	55.3	33: 17	100 × 10^6^ BMMSCs	VAS, WOMAC^1~4^, AE	12 months
Lee, 2018 [25]	Korea	NCT02658344	ADMSC = 12; saline = 12	KL of II, III, or IV; VAS > 4/10	IA of 3 mL	Autologous	62.7	6: 18	100 × 10^6^ ADMSCs	AE	6 months
Lu, 2019 [26]	China	NCT02162693	ADMSC = 26, HA = 26	KL of I, II, or III	IA of 2.5 mL	Autologous	57.3	6: 46	50 × 10^6^ ADMSCs	VAS, WOMAC^1~4^, AE	12 months
Lv, 2015 [27]	China	–	BMMSC = 40; HA = 40	KL of I, II, or III	IA of 5 mL	Autologous	55.5	27: 53	25 × 10^6^ BMMSC	WOMAC^1^, AE	12 months
Matas, 2019 [28]	Chile	NCT02580695	UBSMC = 9; HA = 8	KL of I, II, or III	IA of 3 mL	Allogeneic	55.5	6: 11	20 × 10^6^ UBMSC	VAS, WOMAC^1~4^, AE	12 months
Mendoza, 2017 [19]	Mexico	NCT01485198	BMMSC = 30; CT = 31	KL of II or III	IA of 3 mL	Autologous	57.5	16: 45	20 × 10^6^ BMMSC	VAS, AE	6 months
Vangsness, 2014 [29]	America	NCT00225095	BMMSC = 20; HA = 20	PMM	IA of 5 mL	Allogeneic	46.2	24: 16	20 × 10^6^ BMMSC	AE	24 months
Vega, 2015 [30]	Spain	NCT01586312	BMMSC = 15; HA = 15	KL of II, III, or IV	IA of 3 mL	Allogeneic	57	13: 17	40 × 10^6^ BMMSC	VAS, WOMAC^1~2^, AE	12 months
Wang, 2016 [31]	China	–	UBMSC = 18; HA = 18	KL of II, III, or IV	IA of 3 mL	Allogeneic	53.5	21: 15	20 × 10^6^ UBMSC	WOMAC^1^, AE	6 months

*Abbreviations*: *BMMSC* Bone marrow mesenchymal stem cell, *ADMSC* Adipose-derived mesenchymal stem cell, *UBMSC* Umbilical-derived mesenchymal stem cell, *PRP* Platelet-rich plasma, *HA* Hyaluronic acid, *CT* Conservative treatment, *VAS* visual analog scale, *WOMAC* Western Ontario and McMaster Universities Arthritis Index, *AE* adverse event, *WOMAC*^*1*^ WOMAC total, *WOMAC*^*2*^ WOMAC function, *WOMAC*^*3*^ WOMAC pain, *WOMAC*^*4*^ WOMAC stiffness, *KL* Kellgren and Lawrence system, *NRS* Numerical Rating Scale, *IA* intra-articular injection, *PMM* partial medial meniscectomy

### Risk of bias

**Fig. 2:**
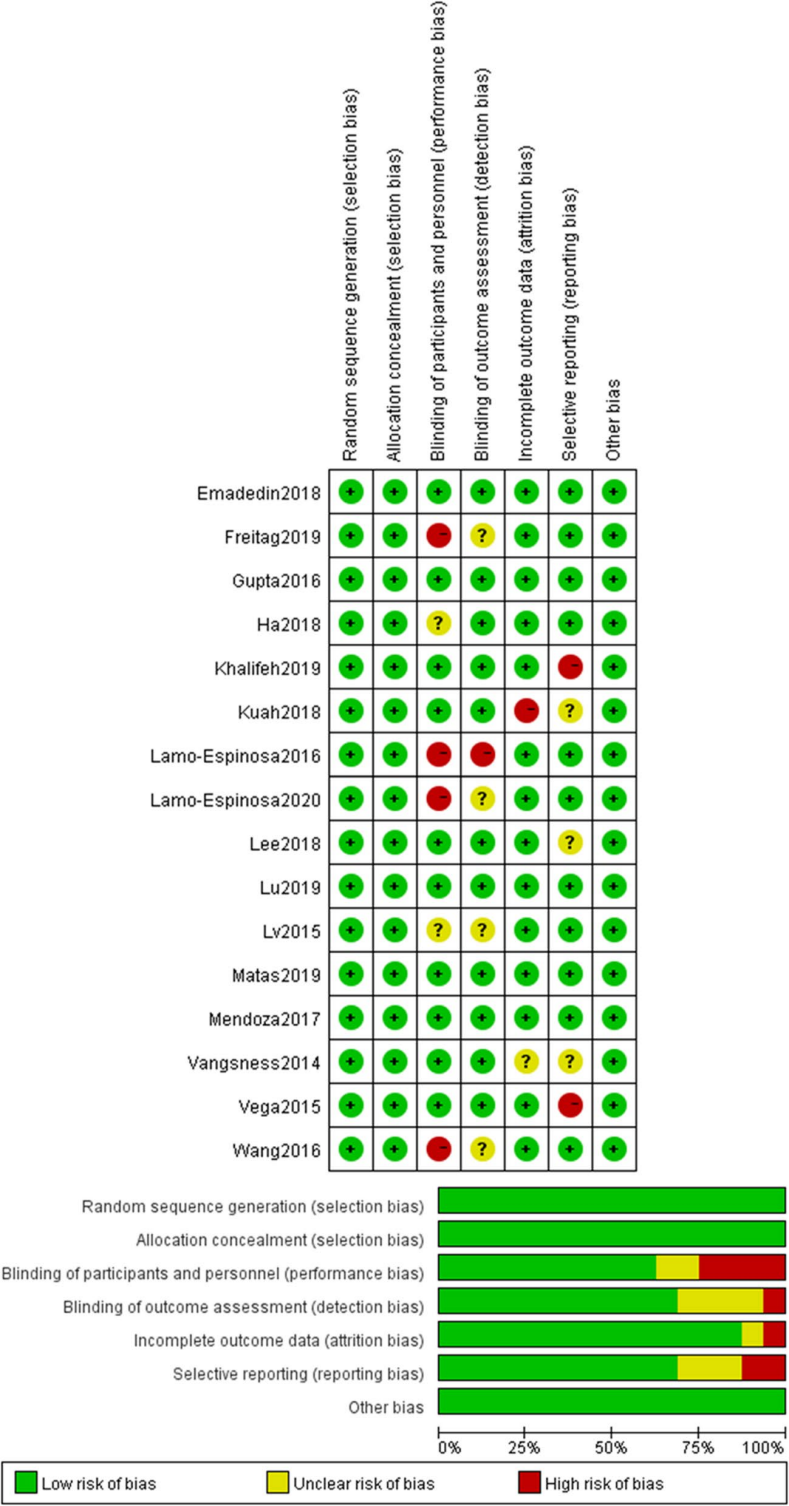
Summary of the risk of bias assessment for the included studies

The results of the Cochrane Risk of Bias Tool in this study showed that 1 study was identified as high risk at two items, and 6 studies were identified as high risk at a single item. Apart from that, most studies showed a low risk of bias at random sequence generation, blinding of participants and personnel, blinding of outcome assessment, incomplete outcome data, and selective reporting, thus indicating a satisfactory quality of the included studies (Fig. 2).

### Pairwise meta-analysis

The results of a direct pairwise meta-analysis showed that intra-articular injection of MSCs was more effective in relieving pain (VAS) than cell-free treatments (ADMSCs vs CT, MD = 1.84, 95% CI − 2.90 to − 0.77; ADMSCs vs HA, MD = − 0.87, 95% CI − 1.44 to − 0.30; ADMSCs vs saline, MD = − 1.52, 95% CI − 2.89 to − 0.15; BMMSCs vs CT, MD = − 2.16, 95% CI − 2.86 to − 1.47; BMMSCs vs HA, MD = − 0.65, 95% CI − 1.13 to − 0.16; BMMSCs vs PRP, MD = − 0.61, 95% CI − 1.17 to − 0.04; UBMSCs vs HA, MD = − 1.08, 95% CI − 2.11 to − 0.06).

The intra-articular injection of MSCs showed better function improvement in WOMAC index totals than the cell-free treatments (ADMSCs vs CT, MD = − 1.70, 95% CI − 2.73 to − 0.66; BMMSCs vs HA, MD = − 0.43, 95% CI − 0.81 to − 0.05; BMMSCs vs saline, MD = − 0.82, 95% CI − 1.44 to − 0.19; UBMSCs vs HA, MD = − 1.74, 95% CI − 2.38 to − 1.10), WOMAC function (UBMSCs vs HA, MD = − 1.27, 95% CI − 2.32 to − 0.21), and WOMAC pain (UBMSCs vs HA, MD = − 1.69, 95% CI − 2.82 to − 0.56).

Based on a pairwise meta-analysis, it was reported that the MSC intra-articular treatments showed a rate of incidence of adverse events but were not significantly different from the rate of incidence in cell-free injection treatments (Additional file 3: Table S1).

### Network meta-analysis

#### Visual analog score (VAS)

**Fig. 3:**
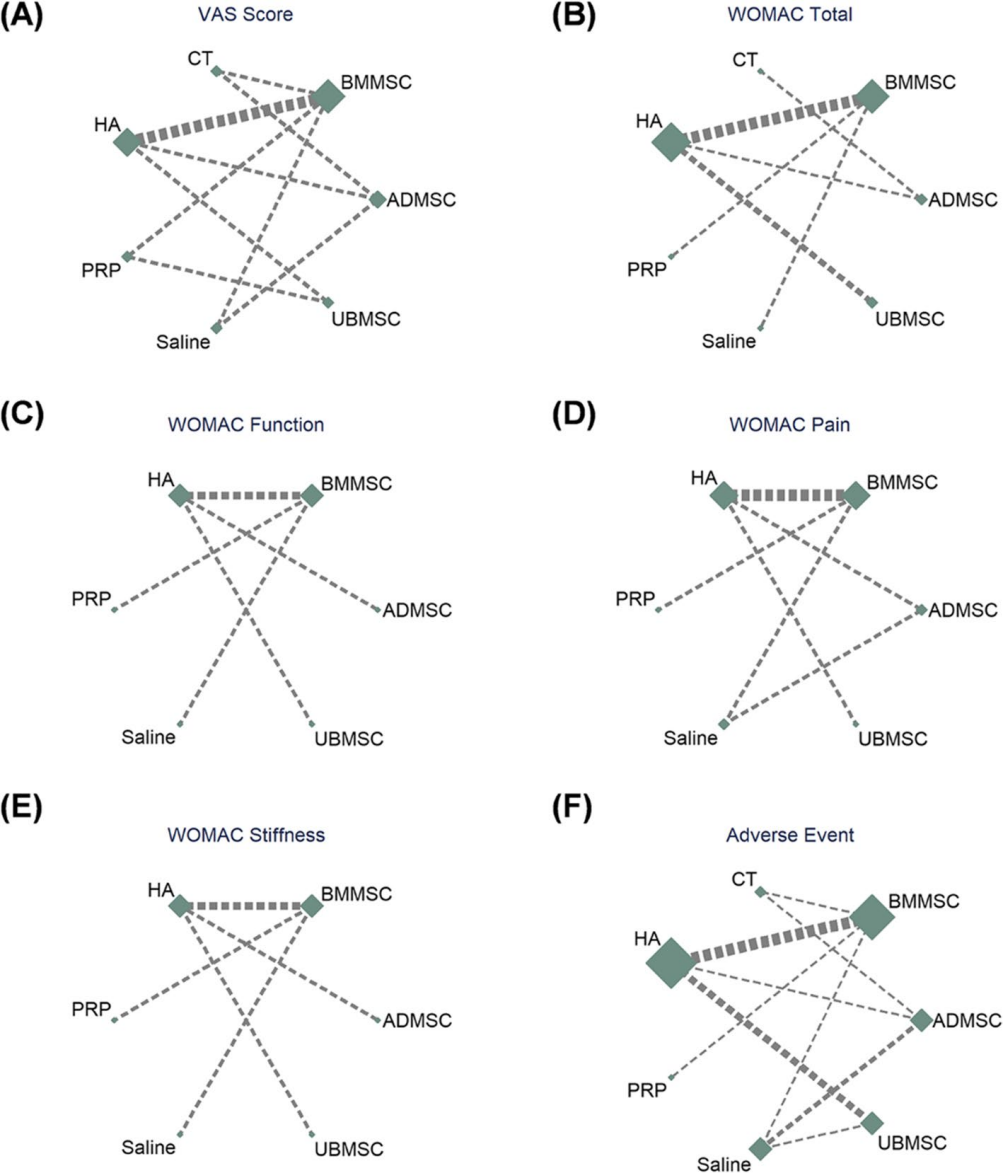
Network geometry of eligible studies. **A** VAS score. **B** WOMAC total. **C** WOMAC function. **D** WOMAC pain. **E** WOMAC stiffness. **F** Adverse events. The size of the node represents the number of patients in each treatment. The thickness of the edges represents the number of contributed studies between the two interventions. VAS, visual analog scale; WOMAC, Western Ontario and McMaster Universities Arthritis Index; BMMSCs, bone marrow mesenchymal stem cell; ADMSCs, adipose mesenchymal stem cell; UBMSCs, umbilical cord mesenchymal stem cell; PRP, platelet-rich plasma; HA, hyaluronic acid; CT, conservative treatment

**Fig. 4:**
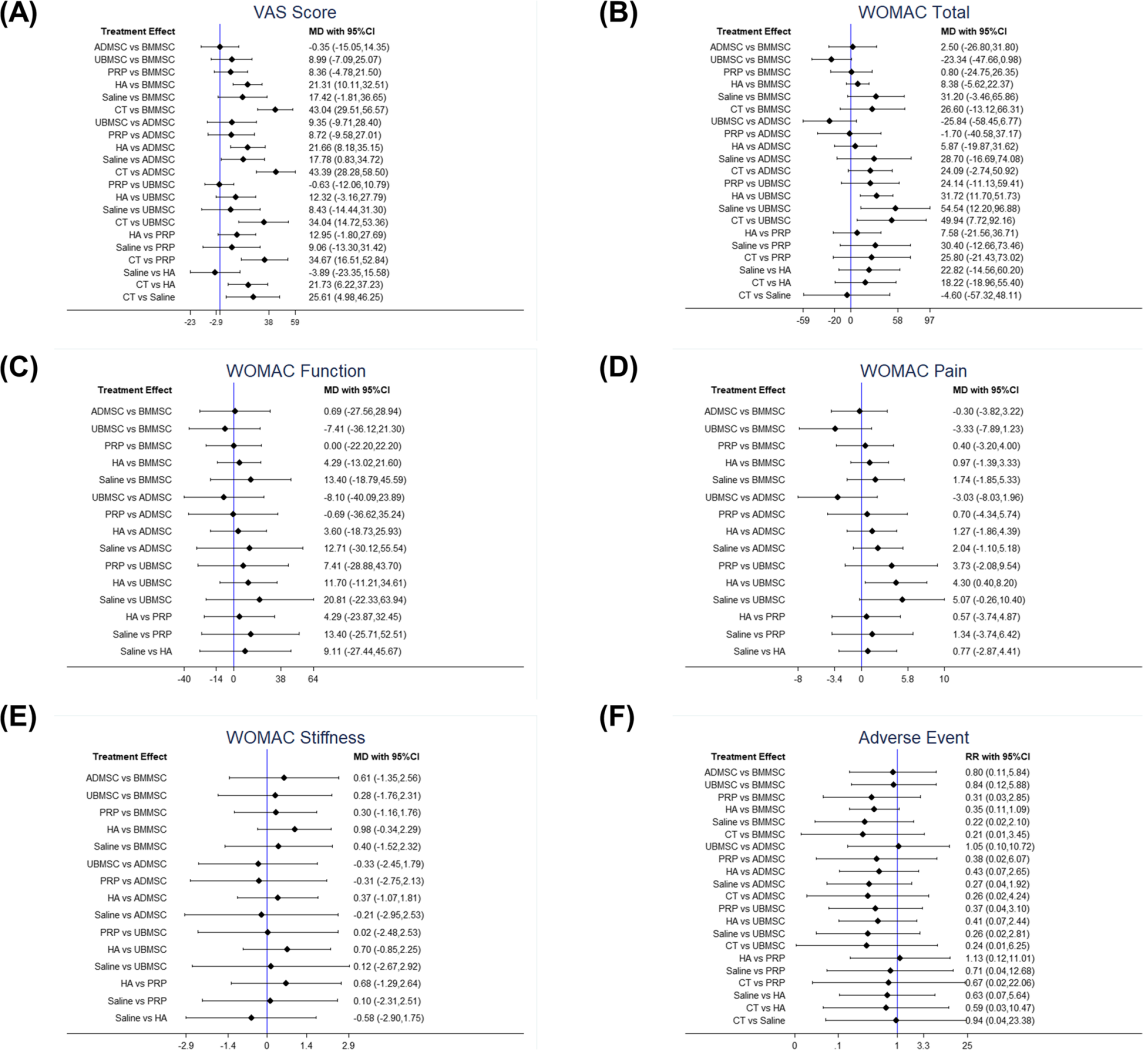
Forest plots of direct comparison between the two interventions. **A** VAS score. **B** WOMAC total. **C** WOMAC function. **D** WOMAC pain. **E** WOMAC stiffness. **F** Adverse events. MD, mean difference; RR, risk ratio

A total of 11 RCTs that comprised 412 patients reported VAS scores between MSC (ADMSC, BMMSC, and UBMSC) and cell-free treatments (PRP, HA, CT, and saline) (Fig. 3A). The intra-articular injection of BMMSCs, ADMSCs, or UBMSCs achieved significantly higher pain relief, VA scores of MD = 43.04, 95% CI 29.51 to 56.57; MD = 43.39, 95% CI 28.28 to 58.50; and MD = 34.04, 95% CI 14.72 to 53.36, respectively, in comparison with CT treatment. A comparative analysis showed that with intra-articular injection of saline, ADMSCs showed remarkable pain improvement, VA score of MD = 17.78, 95% CI 0.83 to 34.72. Furthermore, compared with intra-articular injection of HA, BMMSC and ADMSC treatments yielded pronounced effects on VAS of pain alleviation (MD = 21.31, 95% CI 10.11 to 32.51; MD = 21.66, 95% CI 8.18 to 35.15) (Fig. 4A).

**Fig. 5:**
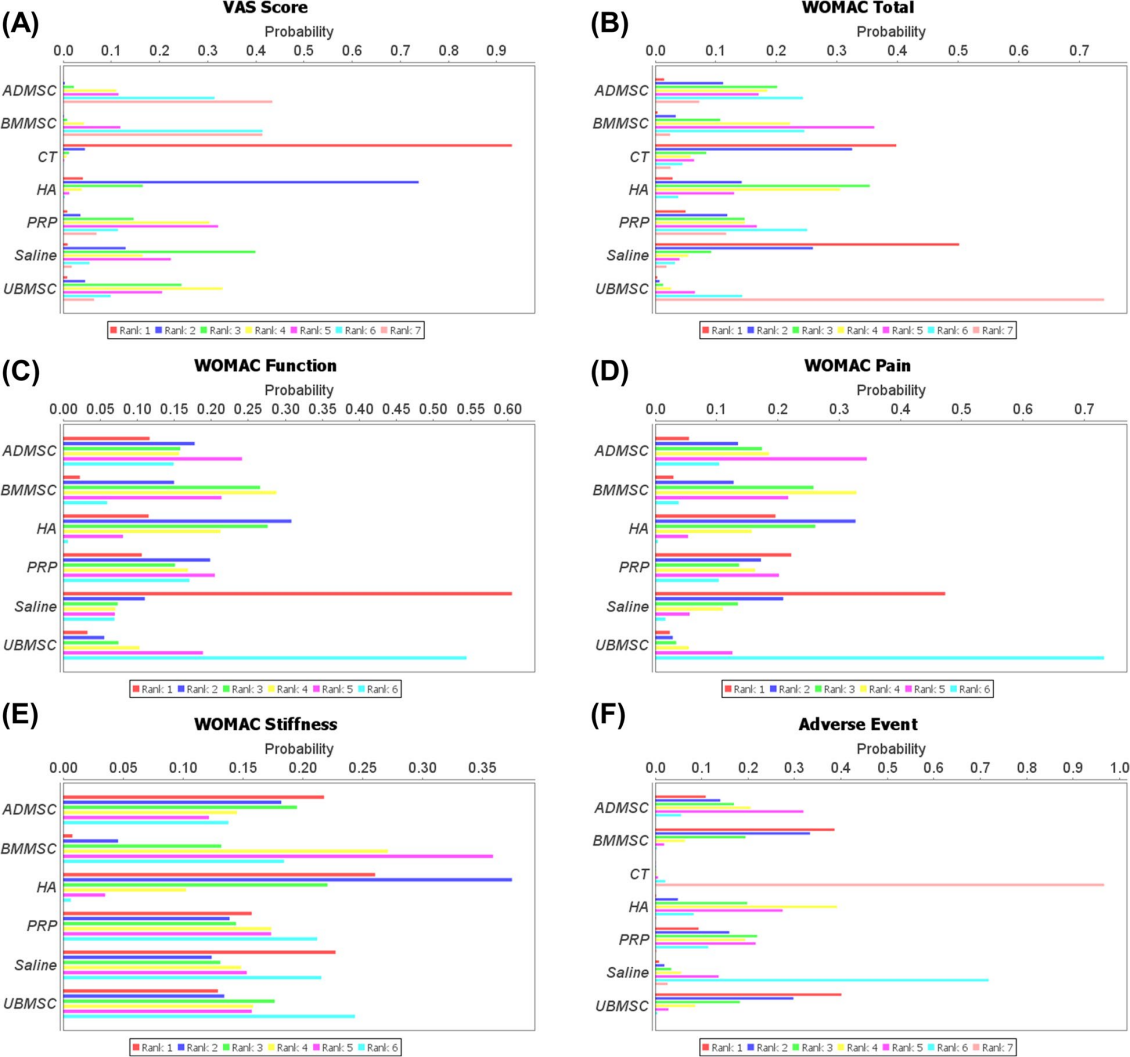
Ranking probability based on the Bayesian model. Different color represents the calculated rank for each intervention (from first to seventh). **A** VAS score. **B** WOMAC total. **C** WOMAC function. **D** WOMAC pain. **E** WOMAC stiffness. **F** Adverse events

**Fig. 6:**
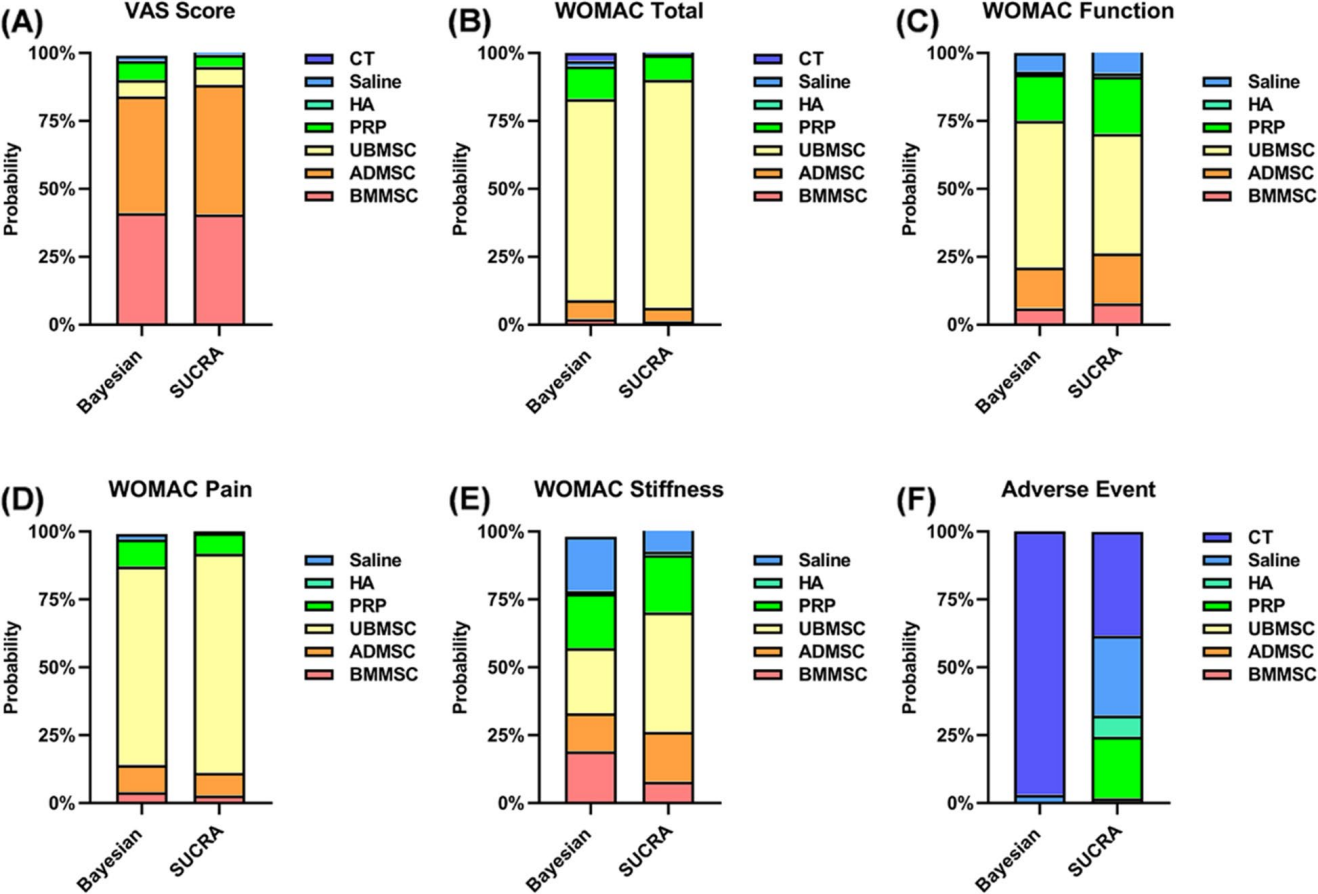
Matching ranking probability of each effect size according to the Bayesian and SUCRA models. Different interventions are represented by different color. **A** VAS score. **B** WOMAC total. **C** WOMAC function. **D** WOMAC pain. **E** WOMAC stiffness. **F** Adverse events

Therefore, according to the Bayesian model, the VAS ranking of the treatments was ADMSC, BMMSC, PRP, UBMSC, saline, HA, and CT (Fig. 5A). According to the SUCRA curve, the VAS ranking of the treatments was ADMSC, BMMSC, UBMSC, PRP, saline, HA, and CT (Fig. S1A). MSC treatments’ dominant ranking probability was found with a good fitting degree for VAS score improvement (Bayesian: 90% versus 10% and SUCRA: 94.9% versus 5.1%) (Fig. 6A).

#### WOMAC total

A total of 10 RCTs that comprised 368 patients reported the WOMAC total between intra-articular injections of MSCs (ADMSCs, BMMSCs, and UBMSCs) and cell-free treatments (PRP, CT, HA, and saline) (Fig. 3B). Intra-articular injection of UBMSCs showed greater improvement in WOMAC total as compared with HA (MD = 31.72, 95% CI 11.70 to 51.73), CT (MD = 49.94, 95% CI 7.72 to 92.16), and saline (MD = 54.54, 95% CI 12.20 to 96.88). (Fig. 4B). The ranking of the treatments based on the Bayesian model was UBMSC, PRP, ADMSC, BMMSC, CT, saline, and HA (Fig. 5B). On the other hand, the ranking of the treatments according to the SUCRA curve was UBMSC, PRP, ADMSC, BMMSC, CE, saline, and HA (Fig. S1B). MSC treatments supported the ranking probability with a good fitting degree for WOMAC total (Bayesian: 83% versus 17% and SUCRA: 90.1% versus 9.9%) (Fig. 6B).

#### WOMAC function

A total of 8 RCTs that comprised 244 patients reported the WOMAC function between MSC treatments (ADMSC, BMMSC, and UBMSC) and cell-free treatments (PRP, HA, and saline) (Fig. 3C). Any two treatments showed insignificantly different. (Fig. 4C). The ranking of the treatments based on the Bayesian model was UBMSC, PRP, ADMSC, BMMSC, saline, and HA (Fig. 5C) while according to the SUCRA curve, the ranking was UBMSC, PRP, ADMSC, BMMSC, saline, and HA (Fig. S1C). The MSC treatments’ favorable ranking probability with a satisfactory fitting degree was found for WOMAC function (Bayesian: 75% versus 25% and SUCRA: 70.2% versus 29.8%) (Fig. 6C).

#### WOMAC pain

A total of 6 RCTs that composed of 202 patients reported the WOMAC pain in the intra-articular injection with MSC treatments (BMMSC, ADMSC, and UBMSC) and cell-free treatments (PRP, HA, and saline) (Fig. 3D). Intra-articular injection with UBMSCs showed more effectiveness in WOMAC pain when compared with HA (MD = 4.3, 95% CI 0.4 to 8.2) (Fig. 4D). The ranking of the treatments was UBMSC, ADMSC, PRP, BMMSC, saline, and HA based on Bayesian model (Fig. 5D) while the ranking of the treatments was UBMSC, ADMSC, PRP, BMMSC, saline, and HA according to the SUCRA curve (Fig. S1D). The WOMAC pain leading ranking probability of the intra-articular injection with MSC treatments was found with an acceptable fitting degree (Bayesian: 87% versus 12% and SUCRA: 91.8% versus 8.2%) (Fig. 6D).

#### WOMAC stiffness

A total of 6 RCTs that comprised 202 patients reported the WOMAC stiffness between MSC treatments (ADMSC, BMMSC, and UBMSC) and cell-free treatments (PRP, HA, and saline) (Fig. 3E). There were insignificant differences observed between any two treatments (Fig. 4E). The ranking of the treatments based on Bayesian model was UBMSC, PRP, ADMSC, BMMSC, saline, and HA (Fig. 5E) while the ranking according to SUCRA curve was UBMSC, PRP, ADMSC, PRP, BMMSC, saline, and HA (Fig. S1E). MSC treatments’ superior ranking probability with a general fitting degree was found for WOMAC stiffness (Bayesian: 57% versus 43% and SUCRA: 70.2% versus 29.8%) (Fig. 6E).

#### Adverse events

A total of 16 RCTs that comprised 612 patients reported adverse events in MSCs (BMMSC, ADMSC, and UBMSC) and cell-free treatments (PRP, HA, saline, and CT) (Fig. 3F). There was no significant difference observed between any two treatments (Fig. 4F). The ranking of the treatments based on Bayesian model was CT, saline, HA, PRP, UBMSC, ADMSC, and BMMSC (Fig. 5F). On the other hand, the ranking of the treatments according to SUCRA curve was CT, saline, HA, PRP, UBMSC, ADMSC, and BMMSC (Fig. S1F). Cell-free treatments’ prominent ranking probability was found with an excellent fitting degree for adverse events incidence (Bayesian: 100% versus 0% and SUCRA: 98.2% versus 1.8%) (Fig. 6F).

#### Heterogeneity and inconsistency test

The loop test was performed on the closed-loop parameters (VAS score, WOMAC pain, and adverse events) to determine the underlying heterogeneity of the above outcomes. It was found that the loop-specific heterogeneity of each loop was less than 5%. This suggests a negligible heterogeneity in our results (Fig. S2). Both the inconsistency model (Fig. S3) and the node-splitting test (Additional file 3: Table S2) showed no significant inconsistency during the determination of inconsistency in closed-loop variables. A credible consistency was noted in the evaluation of inconsistency for open-loop data. Neither the difference between random effects variance and inconsistency variance nor the difference between consistency and inconsistency variance was inconspicuous (Additional file 3: Table S3).

## Discussion

Although surgical therapies such as arthroplasty and osteotomy relieve pain and enhance mobility in osteoarthritis patients, several complications and cost of the interventions limit their application in early and moderate OA patients [32]. On the other hand, arguably less invasive intra-articular injection therapy achieves local enrichment of bioactive chemical compounds and exerts anti-inflammatory and chondroprotective function in the patients [33]. Conventional injectable agents such as HA decrease friction between articular cartilages by mainly acting as a “lube.” Subsequent PRP enhances the biological activity of HA but cannot manifest its long-term therapeutic effect. The latest MSC-based therapy seemly satisfies the needs of cartilage repair via the synergistic action of stem cells and ECM. However, cell-mediated adverse effects such as fever and inflammation hinder its application. To guide the effective clinical treatment of OA, there is an urgent need for several paired studies that compare the efficacy and safety of different injection agents. According to Han et al., HA or steroid injection agents are associated with better outcomes than ADMSC, PRP, or placebo treatments in OA knee joints [34]. Zhao et al. reported that injection with bioactive reagents such as PRP, ADMSCs, and BMMSCs showed superior pain relief and function improvement as compared with HA or saline treatments [35]. A recent meta-analysis study investigating the knee joints in 203 OA patients showed that UBMSCs may be the most effective agent for improving function. Furthermore, this study showed that ADMSCs were the best choice for reducing pain among other agents (BMMSCs, ADMSCs, and UBMSCs) [36]. However, none of the above studies enrolled MSCs and cell-free treatments, and the network analysis was only based on a single model, convinced choice of different interventions is still uncertain.

To overcome these challenges, this study built a comparable network model among BMMSC, ADMSC, UBMSC, PRP, HA, saline, and CT based on a Bayesian random effects model. The model was calibrated according to SUCRA to draw a reliable conclusion. Our results of this study show that intra-articular injections of MSCs are a better pain reliever than cell-free treatment. A parallel tendency was also observed in the functional recovery. The agminated MSCs in the articular cavity of a patient cause a lubricating action and can also release bioactive factors, e.g., transforming growth factor-β, collagen type II, and sulfated glycosaminoglycans. This help regulate the balance between anabolism and catabolism by targeting cartilage, synovium, and subchondral bone [37]. Based on multi-lineage differentiation, in certain circumstance (hypoxia and avascular), MSCs can differentiate into hyaline cartilage to repair the wearing or tearing joints [38]. Meanwhile, MSC-mediated immunomodulatory and anti-inflammatory effects not only inhibit local pain and tension but also provide a suitable atmosphere for cartilage healing [39]. Furthermore, hoarded progenitor or stem cells, other than injected MSCs, are recruited to the injured area and participate in a sustained repair and regeneration process [40]. However, in view of high costs and acquisition challenges, cell-based therapy for patients who have extremely mild symptoms may not be recommended. Conversely, MSC treatments may be more beneficial for patients who are refractory to conservative or oral medicine therapy, which not only alleviates clinical symptoms in a follow-up period, but also encounters the OA progression to delay or avoid joint replacement for moderate or severe patients.

According to our pairwise analyzed results, the cell-based therapy did not increase the risk of adverse events as compared with cell-free treatments. For the network analysis aspect, conservative treatment was associated with an optimum security since no puncture procedure was implemented. Intriguingly, the safety rank of PRP and saline was superior to BMMSCs, ADMSCs, and UBMSCs. This suggests that injected MSCs may result in a higher hazard risk. In combination with pairwise and network results, we considered that enough attention should be paid to MSC treatments in the clinic, though most of the adverse events observed are mild and self-alleviate local reactions without the need for specific intervention. Freitag et al. reported that two patients received a short period of oral prednisolone to treat prolonged effusion after ADMSC injection [18]. Lamo-Espinosa et al. found that 3 participants were required for anti-inflammatory drugs due to articular pain during the first 24 h after infiltration [24]. The above results suggest that MSC therapy-related adverse events should not be skated over. Due to the diversity and heterogeneity of adverse events, previous studies often neglected this indicator. Several previous studies did not perform a pooled analysis, which conceals the potential negative effects [41]. Song et al. integrated 19 studies, and the pooled results indicated that the complication rate after injection was equal between MSCs and cell-free therapy [42]. Jeyaraman et al. also demonstrated that neither BMMSCs nor ADMSCs increased the complication rate based on a small-sample-sized meta-analysis [43]. However, a network meta-analysis carried out by Han et al. showed that intra-articular injection of steroids and HA ranked higher than ADMSCs for the occurrence of adverse events [34]. Zhao et al. reported that both BMMSCs and ADMSCs were associated with a higher incidence of treatment-related adverse events (RR = 3.91 and RR = 8.00, respectively). This further increased the risk introduced by cell treatment in clinical applications [35]. To further explore the potential mechanisms involved in MSC-mediated slight increase in the risk of adverse events, the authors speculated that MSC-associated immunoreactivity, especially allogeneic MSCs, can affect the inflammatory homeostasis in OA joints and contribute to fibrosis or synovitis [9]. For some certain susceptible individuals, the host may recognize the injected MSCs as a foreign matter and subsequently activates the intrinsic defense system to release pro-inflammatory cytokines, e.g., interleukin (IL)-1β and tumor necrosis factor (TNF)-α, and polarize macrophages to inflammation subtypes, e.g., M1 macrophage. Therefore, an efficacious immuno-modulation strategy is indispensable to MSC-based intra-articular injection therapy.

The choice of MSC sources is another crucial concern for cell-based therapy. It was theoretically considered that MSCs exerted a similar therapeutic effect due to their inherent multiple differentiation potency. However, clinical trials showed a discrepant outcome in terms of different MSC types. Wei et al. compared the efficacy of MSCs from different sources and suggested that ADMSCs were the best choice for relieving pain whereas UBMSCs were the most effective method for improving function [36]. According to Ding et al., high dosage of ADMSCs was the most effective treatment among BMMSCs, UBMSCs, and chondrocytes for Knee Injury and Osteoarthritis Outcome Score (KOOS) improvement function and pain relief [44]. Consistently, according to our results, ADMSCs showed a preponderant effect on pain alleviation and UBMSCs acted as an optimum option in function recovery. Considering discrepant chondrogenesis among different MSCs in in vitro pellet culture [45–47], we hypothesized that ADMSCs or UBMSCs may exert a superior anti-arthritic effect via its greater chondrogenic potency rather than osteogenic capacity. For the clinical translational application, apart from therapeutic effect, another factor should be considered carefully: acquisition difficulty. BMMSCs are usually obtained from the iliac crest with great pain and high infection risk. In contrast, ADMSCs that are derived from adipose tissue through liposuction and UBMSCs that are extracted from the postpartum umbilical cord are easier to acquire and expand in vitro [48, 49]. Furthermore, cell sources should be considered with caution. Compared with allogenic MSCs, injection of autologous MSCs may result in slighter immunological rejection and inflammatory reaction. In an experiment equine model, repeated intra-articular injection of allogenic MSCs led to an adverse response rather than the autologous MSCs [50]. Given that autologous UBMSCs are difficult to acquire, engaging our pooled results, autologous ADMSCs may be an optimal choice for treating intractable OA that will be associated with favorable clinical outcomes and negligible immune response.

Several limitations of this study should be noted. First, the study compared three MSC sources with cell-free treatments, but another preclinical method with insufficient data, e.g., synovial mesenchymal stem cells (SMSCs), was not analyzed. Second, additional assessments such as KOOS, Lysholm Knee Scale (Lysholm), and Whole-Organ Magnetic Resonance Imaging Score (WORMS) need to be considered in the future based on more RCTs. Third, due to the relatively short follow-up time, the long-term clinical outcomes of MSC treatments are unclear. Fourth, the follow-up period of enrolled studies was not completely consistent, which may introduce another potential selection bias. We will continue to focus on the therapeutic effects of MSC treatments on OA based on a long-observed period.

## Conclusion

This research analyzed the advantages and limitations of intra-articular injection of three MSCs (ADMSCs, UBMSCs, BMMSCs) for OA patients using a dual network meta-analysis. Our results show that intra-articular administration of MSCs has better pain relief and function improvement than cell-free therapy for treating OA. Furthermore, the study reveals that ADMSCs and UBMSCs are superior to BMMSCs as the MSC sources for OA treatment. However, MSC-based therapy may cause some potential adverse events that need to be paid enough attention to. More studies with large sample sizes and long-term follow-up are required to make conclusive claims in the future.

## Supplementary Information

**Additional file 1: Fig. S1.** Ranking probability based on SUCRA model. Different area under curve represents the predicted rank for each intervention (From first to seventh). (A) VAS score; (B) WOMAC Total; (C) WOMAC Function; (D) WOMAC Pain; (E) WOMAC Stiffness; (F) Adverse events. SUCRA, surface under the cumulative ranking curve. **Fig. S2.** Heterogeneity assessment according to the loop test. (A) VAS score; (B) WOMAC Pain; (C) Adverse events. **Fig. S3.** Inconsistency assessment according to the inconsistency model. (A) VAS score; (B) WOMAC Pain and (C) Adverse events.

**Additional file 2.** PRISMA 2020 Checklist.

**Additional file 3: Table S1.** Pairwise meta-analysis for each outcome based on random effects model. **Table S2.** Local consistency test based on node-splitting method for closed-loop variables. **Table S3.** Consistency test for closed and open-loop data based on the comparison of different model variances.

## Data Availability

All data generated or analyzed during this study are included in this published article and its supplementary information files.

## Abbreviations

OA: Osteoarthritis
MSCs: Mesenchymal stem cells
CNKI: China National Knowledge Internet
RCTs: Randomized controlled trials
ADMSCs: Adipose mesenchymal stem cells
UBMSCs: Umbilical cord mesenchymal stem cells
BMMSCs: Bone marrow mesenchymal stem cells
NSAIDs: Nonsteroidal anti-inflammatory drugs
CS: Corticosteroid
PRP: Platelet-rich plasma
ECM: Extracellular matrix
PRISMA-NMA: Systematic Reviews and Incorporated Network Meta-Analysis
VAS: Visual analog scale
CT: Conservative treatment
WOMAC: Western Ontario and McMaster Universities Osteoarthritis
MCMC: Markov Chains Monte Carlo
KOOS: Knee Injury and Osteoarthritis Outcome Score
SMSCs: Synovial mesenchymal stem cells
WORMS: Whole-Organ Magnetic Resonance Imaging Score
