# Supplementary material for: Intra-articular injection choice for osteoarthritis: making sense of cell source—an updated systematic review and dual network meta-analysis
Source: Arthritis Res Ther. 2022 Nov 28;24:260. doi: 10.1186/s13075-022-02953-0 (PMC9703652; doi:10.1186/s13075-022-02953-0)
Supplement: Supplementary file 1 — Additional file 1: Fig. S1. Ranking probability based on SUCRA model. Different area under curve represents the predicted rank for each intervention (From first to seventh). (A) VAS score; (B) WOMAC Total; (C) WOMAC Function; (D) WOMAC Pain; (E) WOMAC Stiffness; (F) Adverse events. SUCRA, surface under the cumulative ranking curve. Fig. S2. Heterogeneity assessment according to the loop test. (A) VAS score; (B) WOMAC Pain; (C) Adverse events. Fig. S3. Inconsistency assessment according to the inconsistency model. (A) VAS score; (B) WOMAC Pain and (C) Adverse events. [file 13075_2022_2953_MOESM1_ESM.docx]

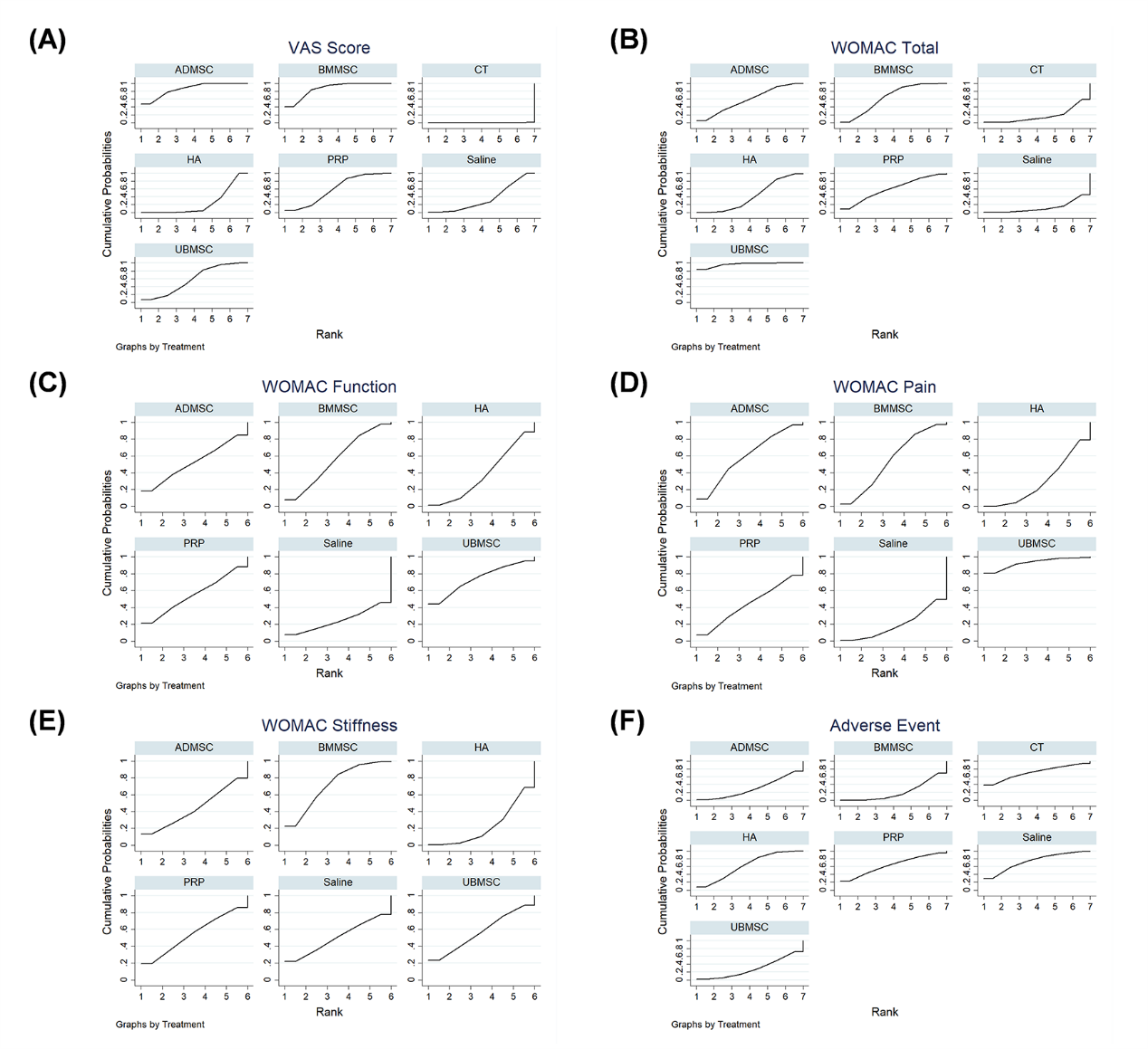


**Supplementary Figure 1:** Ranking probability based on SUCRA model. Different area under curve represents the predicted rank for each intervention (From first to seventh). (A) VAS score; (B) WOMAC Total; (C) WOMAC Function; (D) WOMAC Pain; (E) WOMAC Stiffness; (F) Adverse events. SUCRA, surface under the cumulative ranking curve.

**
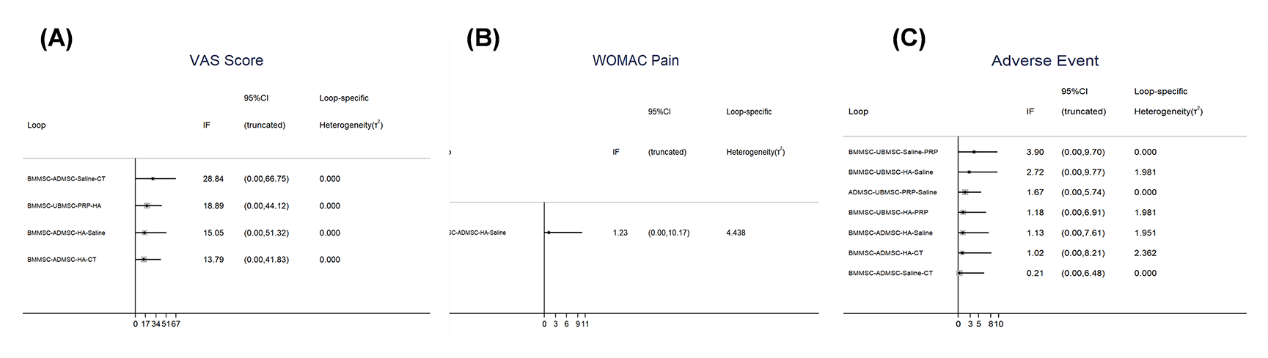
**

**Supplementary Figure 2:** Heterogeneity assessment according to the loop test. (A) VAS score; (B) WOMAC Pain; (C) Adverse events.

**
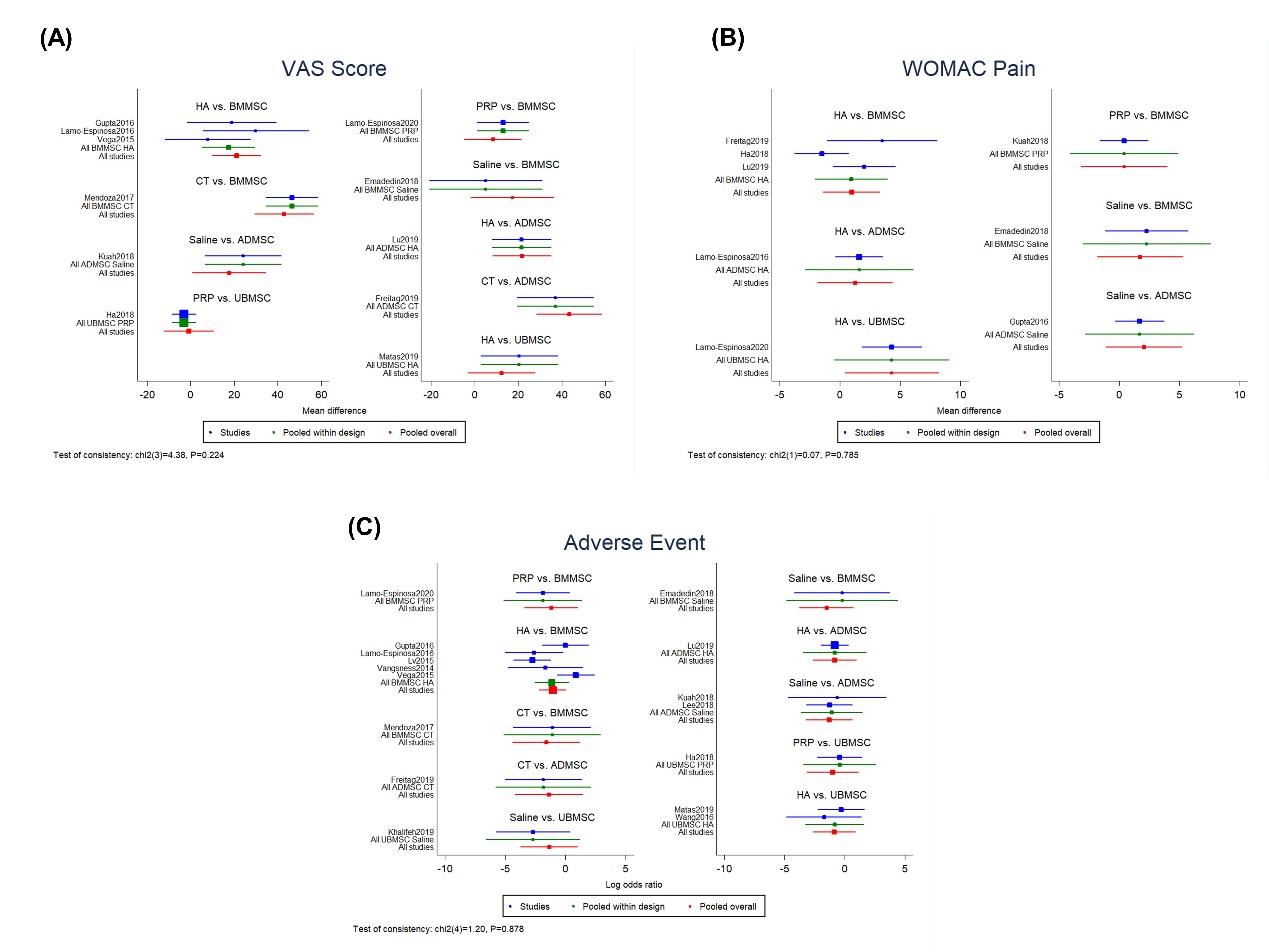
**

**Supplementary Figure 3:** Inconsistency assessment according to the inconsistency model. (A) VAS score; (B) WOMAC Pain and (C) Adverse events.
