# Supplementary material for: Intra-articular injection choice for osteoarthritis: making sense of cell source—an updated systematic review and dual network meta-analysis
Source: Arthritis Res Ther. 2022 Nov 28;24:260. doi: 10.1186/s13075-022-02953-0 (PMC9703652; doi:10.1186/s13075-022-02953-0)
Supplement: Supplementary file 3 — Additional file 3: Table S1. Pairwise meta-analysis for each outcome based on random effects model. Table S2. Local consistency test based on node-splitting method for closed-loop variables. Table S3. Consistency test for closed and open-loop data based on the comparison of different model variances. [file 13075_2022_2953_MOESM3_ESM.docx]

**Supplementary Table 1.** Pairwise meta-analysis for each outcome based on random effects model.

| VAS Score | | | | | | |
| --- | --- | --- | --- | --- | --- | --- |
| ADMSC |  |  |  |  |  |  |
| NA | BMMSC |  |  |  |  |  |
| **-1.84 (-2.90, -0.77)** | **-2.16 (-2.86, -1.47)** | CT |  |  |  |  |
| **-0.87 (-1.44, -0.30)** | **-0.65 (-1.13, -0.16)** | NA | HA |  |  |  |
| NA | **-0.61 (-1.17, -0.04)** | NA | NA | PRP |  |  |
| **-1.52 (-2.89, -0.15)** | -0.12 (-0.72, 0.48) | NA | NA | NA | Saline |  |
| NA | NA | NA | **-1.08 (-2.11, -0.06)** | 0.23 (-0.19, 0.65) | NA | UBMSC |
| WOMAC Total |  |  |  |  |  |  |
| ADMSC |  |  |  |  |  |  |
| NA | BMMSC |  |  |  |  |  |
| **-1.70 (-2.73, -0.66)** | NA | CT |  |  |  |  |
| -0.32 (-0.87, 0.23) | **-0.43 (-0.81, -0.05)** | NA | HA |  |  |  |
| NA | -0.05 (-0.60, 0.51) | NA | NA | PRP |  |  |
| NA | **-0.82 (-1.44, -0.19)** | NA | NA | NA | Saline |  |
| NA | NA | NA | **-1.74 (-2.38, -1.10)** | NA | NA | UBMSC |
| WOMAC Function |  |  |  |  |  |  |
| ADMSC |  |  |  |  |  |  |
| NA | BMMSC |  |  |  |  |  |
| -0.27 (-0.82, 0.28) | -0.24 (-1.41, 0.92) | HA |  |  |  |  |
| NA | 0.00 (-0.55, 0.55) | NA | PRP |  |  |  |
| NA | -0.35 (-0.96, 0.26) | NA | NA | Saline |  |  |
| NA | NA | **-1.27 (-2.32, -0.21)** | NA | NA | UBMSC |  |
| WOMAC Pain |  |  |  |  |  |  |
| ADMSC |  |  |  |  |  |  |
| NA | BMMSC |  |  |  |  |  |
| -0.44 (-0.99, 0.11) | -0.23 (-0.98, 0.52) | HA |  |  |  |  |
| NA | -0.11 (-0.67, 0.44) | NA | PRP |  |  |  |
| -0.86 (-2.12, 0.39) | -0.43 (-1.04, 0.18) | NA | NA | Saline |  |  |
| NA | NA | **-1.69 (-2.82, -0.56)** | NA | NA | UBMSC |  |
| WOMAC Stiffness |  |  |  |  |  |  |
| ADMSC |  |  |  |  |  |  |
| NA | BMMSC |  |  |  |  |  |
| -0.20 (-0.75, 0.34) | -0.63 (-1.27, 0.01) | HA |  |  |  |  |
| NA | -0.17 (-0.72, 0.39) | NA | PRP |  |  |  |
| NA | -0.12 (-0.72, 0.49) | NA | NA | Saline |  |  |
| NA | NA | -0.59 (-1.57, 0.38) | NA | NA | UBMSC |  |
| Adverse Event |  |  |  |  |  |  |
| ADMSC |  |  |  |  |  |  |
| NA | BMMSC |  |  |  |  |  |
| 5.00 (0.27, 92.62) | 2.89 (0.12, 67.75) | CT |  |  |  |  |
| 1.36 (0.89, 2.08) | 1.56 (0.73, 3.30) | NA | HA |  |  |  |
| NA | 5.42 (0.68, 43.10) | NA | NA | PRP |  |  |
| 1.43 (0.83, 2.45) | NA | NA | NA | NA | Saline |  |
| NA | NA | NA | 1.43 (0.49, 4.20) | 1.53 (0.27, 8.74) | 9.00 (0.55, 147.95) | UBMSC |

**Supplementary Table 2.** Local consistency test based on node-splitting method for closed-loop variables.

| VAS Score | Direct Effect | Indirect Effect | Overall | *p* value |
| --- | --- | --- | --- | --- |
| ADMSC, CT | 37.27 (3.03, 68.78) | 50.22 (11.23, 92.99) | 43.20 (21.90, 64.11) | 0.51 |
| ADMSC, HA | 21.40 (-10.43, 52.95) | 30.10 (-6.10, 68.87) | 24.50 (6.43, 44.60) | 0.64 |
| ADMSC, Saline | 23.53 (-1.65, 49.57) | -0.64 (-31.39, 30.63) | 14.07 (-5.91, 34.88) | 0.18 |
| BMMSC, CT | 46.27 (18.16, 77.23) | 32.84 (-10.49, 73.16) | 43.31 (22.22, 61.21) | 0.50 |
| BMMSC, HA | 24.76 (-0.37, 48.49) | 25.88 (-12.21, 58.05) | 24.84 (7.75, 41.04) | 0.97 |
| BMMSC, PRP | 13.41 (-14.66, 41.60) | 0.12 (-48.57, 43.79) | 9.43 (-10.64, 29.79) | 0.44 |
| BMMSC, Saline | 7.57 (-14.58, 28.97) | 32.56 (-3.56, 67.51) | 13.95 (-4.88, 31.87) | 0.18 |
| HA, UBMSC | -20.14 (-50.09, 8.95) | -6.14 (-49.62, 37.61) | -14.04 (-36.31, 7.04) | 0.43 |
| PRP, UBMSC | 2.67 (-24.44, 32.53) | -10.60 (-59.65, 37.99) | 1.60 (-19.04, 18.86) | 0.54 |
| ADMSC, CT | 37.27 (3.03, 68.78) | 50.22 (11.23, 92.99) | 43.20 (21.90, 64.11) | 0.51 |
| ADMSC, HA | 21.40 (-10.43, 52.95) | 30.10 (-6.10, 68.87) | 24.50 (6.43, 44.60) | 0.64 |
| WOMAC Pain | Direct Effect | Indirect Effect | Overall | P-Value |
| ADMSC, HA | 1.58 (-4.14, 7.33) | 0.11 (-8.47, 9.58) | 1.36 (-3.07, 5.75) | 0.76 |
| ADMSC, Saline | 1.73 (-3.94, 7.33) | 2.90 (-6.08, 12.08) | 2.05 (-2.45, 6.40) | 0.80 |
| BMMSC, HA | 0.90 (-2.44, 4.69) | 2.22 (-8.32, 12.62) | 0.91 (-1.82, 4.34) | 0.76 |
| BMMSC, Saline | 2.23 (-3.76, 8.46) | 0.74 (-7.50, 9.89) | 1.64 (-2.85, 6.50) | 0.74 |
| Adverse Event | Direct Effect | Indirect Effect | Overall | P-Value |
| ADMSC, CT | -32.29 (-96.50, -1.97) | -29.19 (-92.00, 1.27) | -29.02 (-77.46, -2.17) | 0.90 |
| ADMSC, HA | -0.91 (-4.77, 2.86) | 9.61 (-2.43, 35.31) | -0.18 (-3.60, 3.56) | 0.13 |
| ADMSC, Saline | -1.33 (-5.53, 2.71) | -18.92 (-63.96, -0.59) | -2.32 (-6.13, 1.26) | 0.08 |
| BMMSC, CT | -17.09 (-63.33, 0.60) | -26.61 (-103.78, -2.64) | -30.43 (-78.94, -3.68) | 0.72 |
| BMMSC, HA | -1.44 (-3.62, 0.45) | -3.71 (-10.84, 2.88) | -1.63 (-3.64, 0.11) | 0.49 |
| BMMSC, PRP | -2.26 (-7.37, 2.02) | 0.12 (-5.66, 6.29) | -1.27 (-4.75, 2.16) | 0.48 |
| BMMSC, Saline | -3.73 (-83.09, 48.75) | -3.79 (-9.39, 0.48) | -3.74 (-8.88, 0.66) | 1.00 |
| HA, UBMSC | 1.61 (-1.75, 5.64) | 2.11 (-3.26, 8.34) | 1.65 (-0.93, 4.93) | 0.88 |
| PRP, UBMSC | 0.52 (-3.83, 4.99) | 3.07 (-2.79, 9.65) | 1.34 (-1.97, 4.89) | 0.48 |
| Saline, UBMSC | 18.61 (1.71, 48.97) | 1.67 (-4.62, 8.27) | 3.74 (-0.18, 9.34) | 0.09 |

**Supplementary Table 3.** Consistency test for closed and open-loop data based on the comparison of different model variances.

| VAS Score |  |  |  |
| --- | --- | --- | --- |
| The first test | Median (95% CrI) | The second test | Median (95% CrI) |
| Random Effects Standard Deviation from consistence model | 6.27 (0.74, 25.61) | Random Effects Standard Deviation from inconsistence model | 6.41 (0.53, 29.37) |
| Random Effects Standard Deviation from inconsistence model | 6.41 (0.53, 29.37) | Inconsistency Standard Deviation from inconsistency model | 15.23 (1.49, 43.34) |
| WOMAC Total |  |  |  |
| The first test | Median (95% CrI) | The second test | Median (95% CrI) |
| Random Effects Standard Deviation from consistence model | 15.99 (4.95, 43.49) | Random Effects Standard Deviation from inconsistence model | 15.81 (4.53, 42.80) |
| Random Effects Standard Deviation from inconsistence model | 15.81 (4.53, 42.80) | Inconsistency Standard Deviation from inconsistency model | 25.57 (1.20, 49.66) |
| WOMAC Function |  |  |  |
| The first test | Median (95% CrI) | The second test | Median (95% CrI) |
| Random Effects Standard Deviation from consistence model | 8.54 (0.90, 13.83) | Random Effects Standard Deviation from inconsistence model | 8.37 (0.99, 13.81) |
| Random Effects Standard Deviation from inconsistence model | 8.37 (0.99, 13.81) | Inconsistency Standard Deviation from inconsistency model | 7.06 (0.35, 13.74) |
| VAS Pain |  |  |  |
| The first test | Median (95% CrI) | The second test | Median (95% CrI) |
| Random Effects Standard Deviation from consistence model | 1.94 (0.25, 4.09) | Random Effects Standard Deviation from inconsistence model | 1.98 (0.16, 4.11) |
| Random Effects Standard Deviation from inconsistence model | 1.98 (0.16, 4.11) | Inconsistency Standard Deviation from inconsistency model | 1.91 (0.06, 4.17) |
| WOMAC Stiffness |  |  |  |
| The first test | Median (95% CrI) | The second test | Median (95% CrI) |
| Random Effects Standard Deviation from consistence model | 0.87 (0.02, 1.75) | Random Effects Standard Deviation from inconsistence model | 0.86 (0.09, 1.75) |
| Random Effects Standard Deviation from inconsistence model | 0.86 (0.09, 1.75) | Inconsistency Standard Deviation from inconsistency model | 0.90 (0.04, 1.76) |
| Adverse Event |  |  |  |
| The first test | Median (95% CrI) | The second test | Median (95% CrI) |
| Random Effects Standard Deviation from consistence model | 1.75 (0.64, 2.62) | Random Effects Standard Deviation from inconsistence model | 1.78 (0.67, 2.62) |
| Random Effects Standard Deviation from inconsistence model | 1.78 (0.67, 2.62) | Inconsistency Standard Deviation from inconsistency model | 1.27 (0.11, 2.59) |
